# Radioactive Beams for Image-Guided Particle Therapy: The BARB Experiment at GSI

**DOI:** 10.3389/fonc.2021.737050

**Published:** 2021-08-19

**Authors:** Daria Boscolo, Daria Kostyleva, Mohammad Javad Safari, Vasiliki Anagnostatou, Juha Äystö, Soumya Bagchi, Tim Binder, Georgios Dedes, Peter Dendooven, Timo Dickel, Vasyl Drozd, Bernhard Franczack, Hans Geissel, Chiara Gianoli, Christian Graeff, Tuomas Grahn, Florian Greiner, Emma Haettner, Roghieh Haghani, Muhsin N. Harakeh, Felix Horst, Christine Hornung, Jan-Paul Hucka, Nasser Kalantar-Nayestanaki, Erika Kazantseva, Birgit Kindler, Ronja Knöbel, Natalia Kuzminchuk-Feuerstein, Bettina Lommel, Ivan Mukha, Chiara Nociforo, Shunki Ishikawa, Giulio Lovatti, Munetaka Nitta, Ikechi Ozoemelam, Stephane Pietri, Wolfgang R. Plaß, Andrej Prochazka, Sivaji Purushothaman, Claire-Anne Reidel, Heidi Roesch, Fabio Schirru, Christoph Schuy, Olga Sokol, Timo Steinsberger, Yoshiki K. Tanaka, Isao Tanihata, Peter Thirolf, Walter Tinganelli, Bernd Voss, Uli Weber, Helmut Weick, John S. Winfield, Martin Winkler, Jianwei Zhao, Christoph Scheidenberger, Katia Parodi, Marco Durante, the Super-FRS Experiment Collaboration

**Affiliations:** ^1^GSI Helmholtzzentrum für Schwerionenforschung, Darmstadt, Germany; ^2^Ludwig-Maximilians-Universität München, Munich, Germany; ^3^University of Jyväskylä, Jyväskylä, Finland; ^4^Helsinki Institute of Physics, Helsinki, Finland; ^5^Indian Institute of Technology, Dhanbad, India; ^6^University Medical Center Groningen, Groningen, Netherlands; ^7^Justus-Liebig-Universität Gießen, Gießen, Germany; ^8^University of Groningen, Groningen, Netherlands; ^9^Technische Universität Darmstadt, Darmstadt, Germany; ^10^Tohoku University, Sendai, Japan; ^11^Fontys University of Applied Sciences, Eindhoven, Netherlands; ^12^MedAustron, Wiener Neustadt, Austria; ^13^RIKEN High Energy Nuclear Physics Laboratory, Wako, Japan; ^14^Research Center for Nuclear Physics, Osaka University, Osaka, Japan; ^15^Peking University, Beijing, China; ^16^Institute of Modern Physics, Lanzhou, China

**Keywords:** particle therapy, radioactive ion beams, carbon ions, oxygen ions, PET

## Abstract

Several techniques are under development for image-guidance in particle therapy. Positron (β^+^) emission tomography (PET) is in use since many years, because accelerated ions generate positron-emitting isotopes by nuclear fragmentation in the human body. In heavy ion therapy, a major part of the PET signals is produced by β^+^-emitters generated *via* projectile fragmentation. A much higher intensity for the PET signal can be obtained using β^+^-radioactive beams directly for treatment. This idea has always been hampered by the low intensity of the secondary beams, produced by fragmentation of the primary, stable beams. With the intensity upgrade of the SIS-18 synchrotron and the isotopic separation with the fragment separator FRS in the FAIR-phase-0 in Darmstadt, it is now possible to reach radioactive ion beams with sufficient intensity to treat a tumor in small animals. This was the motivation of the BARB (Biomedical Applications of Radioactive ion Beams) experiment that is ongoing at GSI in Darmstadt. This paper will present the plans and instruments developed by the BARB collaboration for testing the use of radioactive beams in cancer therapy.

## Introduction

Image-guidance is one of the major improvements of radiotherapy in the past years (1). High resolution imaging allows dose escalation, hypofractionation, and treatment of moving tumors with tracking (2). Image-guided particle therapy is currently less mature, even if the problem of range uncertainty is a major caveat compared to conventional radiotherapy (3). Range uncertainty in the patient is typically compensated by using wide target margins: in proton therapy, the margin is about 3.5% of the prescribed range (4). The widening of the margins jeopardizes one of the main advantages of the Bragg peak: the steep distal dose gradients and the potentially high targeting accuracy and precision (5). Actually, the physics of particle therapy offers several imaging methods that are ruled out in photon therapy (6). For instance, prompt γ-rays (PG) generated in nuclear reactions can be detected and the signal fall-off is correlated to the Bragg peak (7). In heavy ion therapy it is also possible to measure the range by detecting secondary charged particles, such as protons emitted at large angles (8, 9). A combination of different methods is under study for animal irradiators (10) and in clinical settings (11, 12).

The range verification method that has been tested most extensively in clinical practice is positron emission tomography (PET) (13). Unlike conventional diagnostic imaging (14), PET in particle therapy exploits β^+^-emitting isotopes produced by the particle beam in the patient’s body by nuclear fragmentation. In proton therapy, only target fragments can be used for PET imaging, while in heavy ion therapy the projectile fragments provide a large part of the PET signal with better correlation to the dose. Because the time of flight of the ions in the patient is much smaller than the half-life of the β^+-^emitters, the positron emission occurs essentially after the fast fragments are stopped in tissue. For instance, ^12^C-ions, used in a dozen centers worldwide for cancer therapy (15), produce positron emitting ^11^C (t_1/2 _= 20.3 _min_) and ^10^C (t_1/2 =_ 19.3 s) nuclei by nuclear fragmentation. The peak in the activity from the isotopic projectile fragments is visualized upstream of the Bragg peak, because such fragments, lighter than the projectile, have shorter ranges at the same velocity of the primary ion (16, 17). Online PET was used for the first time clinically during the ^12^C-ion pilot therapy project at GSI, Darmstadt, 1997-2008 (18) and a number of particle therapy centers are currently using this technique for range verification off-line (12, 19–21).

However, PET in ^12^C-ion therapy remains marginal and not really able to reduce the range uncertainty as desired. The half-life of the most abundant induced radionuclides is too long for instantaneous feedback while the short-lived radionuclides are produced at a very low rate and exhibit a long positron range (22) before annihilation. The physical shift in the β^+^-activity and ^12^C dose peaks along with the biological washout requires Monte Carlo (MC) simulations (23) or other analytical calculations (24) currently unavoidable for data analysis. Eventually, the low counting rate of β^+^-emitting fragments and the uncertainties in MC calculations limit the accuracy of PET-based range verification to about 2-5 mm (6, 19, 25).

**Figure 1:**
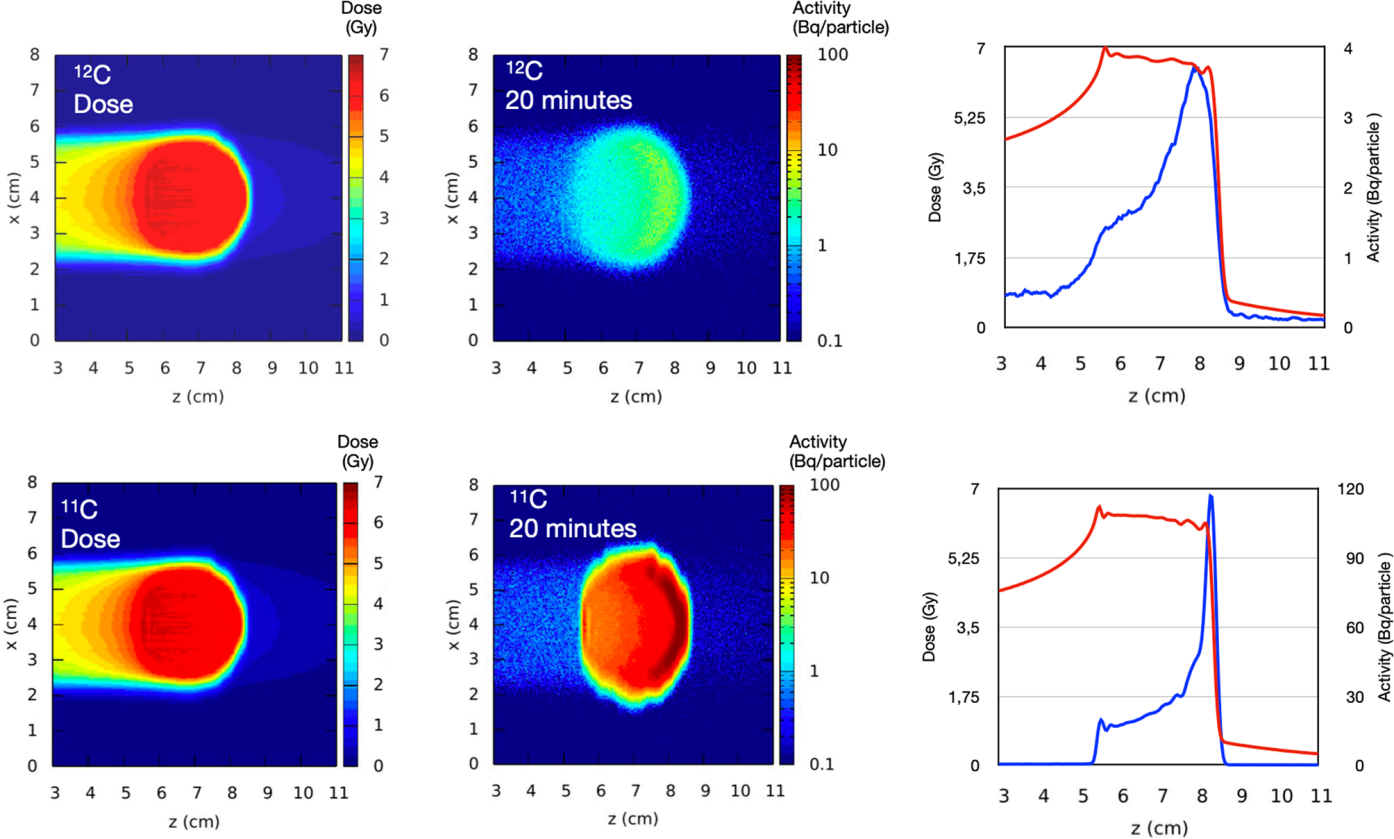
Monte Carlo simulation of ^12^C and ^11^C beams stopping in a spherical water volume and visualized by PET in 20 min. The graphs show the dose (red curve) and the activity (blue curve) distribution along the beam direction (z-axis) showing the shift between dose and activity when stable ions are used. Simulation by Monte Carlo code FLUKA.

Most of these problems are automatically overcome if β^+^-radioactive ion beams (RIB) are directly used for both treatment and imaging. Such radioactive ion beams would improve the count rate by an order of magnitude (26), reduce the shift between measured activity and dose (27), and mitigate the washout blur of the image with short-lived isotopes and in-beam acquisition, eventually leading to sub-mm resolution. Attempts to use RIB in therapy started almost half a century ago during the heavy ion therapy pilot project at the Lawrence Berkeley Laboratory (CA, USA) (28), but they were always hampered by the low intensity of the secondary beams produced by fragmentation of the primary ion used for therapy (29). New, high-intensity accelerators can produce radioactive ion beams with an intensity sufficient for therapeutic treatment (30), and this would pave the way to PET-guided heavy ion treatment. The advantages of using RIB for simultaneous treatment and imaging in comparison to conventional PET imaging in C-ion therapy are shown in the Monte Carlo simulation in **Figure 1**.

The recent upgrade of the SIS-18 accelerator at GSI (Darmstadt, Germany) toward the construction of FAIR (31), i.e. the so-called FAIR-phase-0 (32), gives the opportunity to resume early studies with PET imaging of RIB at GSI (33) and test its application *in vivo*. The BARB (Biomedical Applications of Radioactive ion Beams) project, funded by the European Research Council (ERC) in 2020, aims at treating a tumor in mouse “patients” with RIB (^11^C and ^15^O) with an imaging resolution around 0.5 mm. To this goal, within BARB the Ludwig-Maximilians-Universität (LMU) Munich will develop an innovative hybrid detector, able to exploit the PG emission during the synchrotron spill delivery in the target, and counting PET signals in-between the synchrotron spills (34). In this paper, we will present the planned experiments and the technologies that will be developed and applied in BARB to reach the goal of *in vivo* tumor treatment with RIB.

## The Fragment Separator FRS

**Figure 2:**
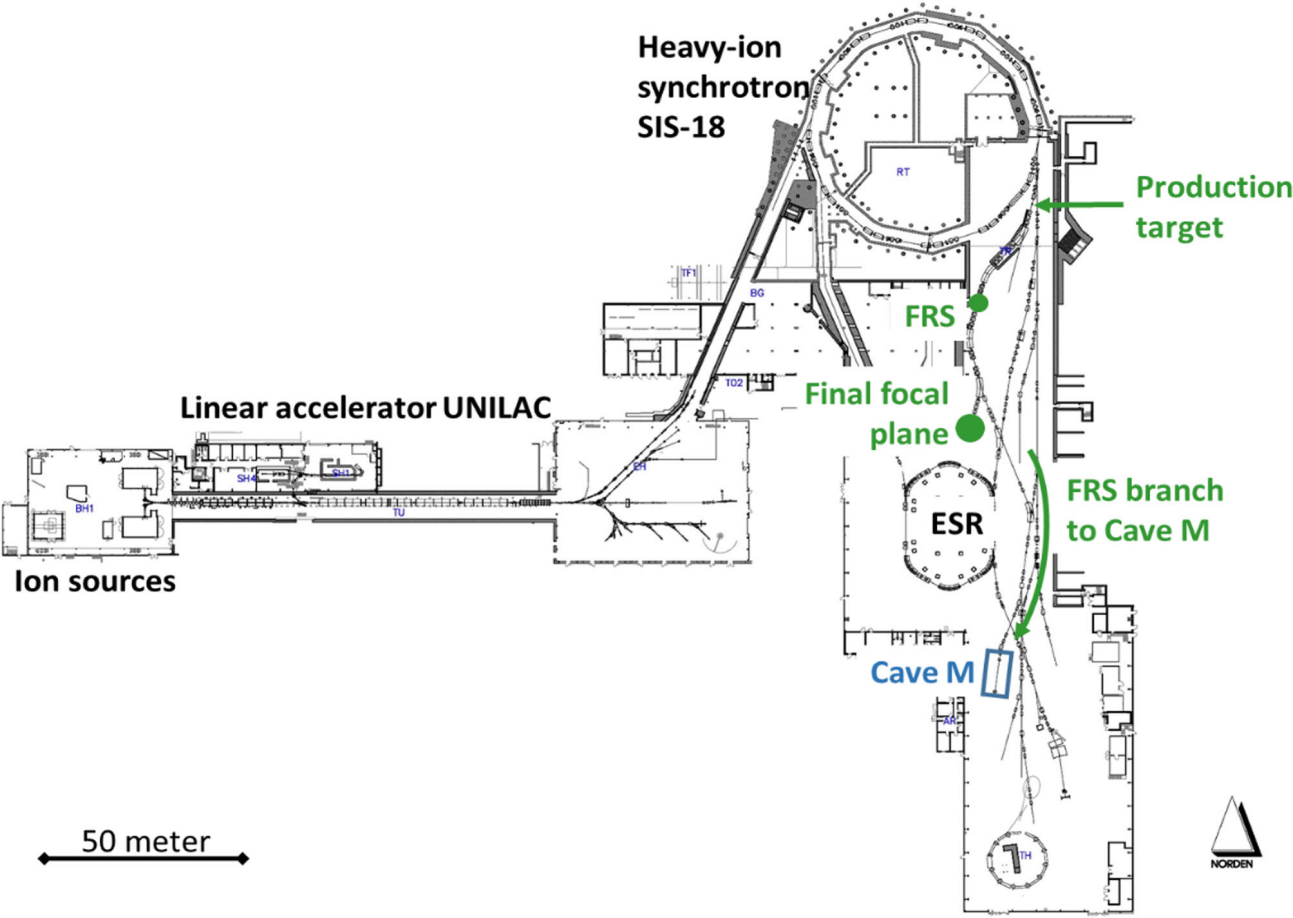
Overview of the existing GSI-FAIR accelerator facility UNILAC-SIS-ESR. The FRS is the radioactive beam facility of GSI and a high-resolution magnetic spectrometer that provides a large variety of secondary nuclear beams ranging from hydrogen up to uranium, among them ^10,11^C or ^14,15^O, which can be produced from intense primary beams (like ^12^C or ^16^O) in the production target at its entrance. The ions of interest are identified in flight, spatially separated, energy bunched and used for experimental studies at the FRS itself (central and final focal plane, respectively, indicated by green dots) or they are transmitted *via* the FRS branch to the target hall for experiments in Cave M.

The GSI-FAIR accelerators provide intense primary beams of all chemical elements from hydrogen up to uranium and their energies range from a few keV/u up to the relativistic regime. For instance, the heavy ion synchrotron SIS-18 can accelerate protons up to 4.5 GeV, and 2,000 MeV/u can be reached for beams with a mass-to-charge ratio A/Z=2, corresponding to 18 Tm magnetic rigidity. At these energies, light ions like C, O or Ne attain ranges of many centimeters in matter, e.g. in water. At the fragment separator FRS (35), the stable ion beams undergo nuclear reactions in a production target located at its entrance and produce a large variety of secondary beams. These fragments are kinematically focused in forward direction and have velocities that are similar to the primary ions. Due to the nuclear reaction kinematics and atomic effects (such as energy loss, energy-loss straggling and multiple angular scattering) in the production target, the fragments have a large transverse and longitudinal phase-space, much larger than the primary beam. In particular, the relative momentum spread Δp/p is of the order of a few percent, compared to the primary beam with 5×10^-4^. This leads consequently to a drastically larger range straggling that is a Bragg Peak with a width of several millimeters when the fragments are stopped in matter. If needed, this range spread can be reduced by combining the dispersive magnetic system of FRS with shaped degraders, which reduces the energy and range straggling of the fragment beams down to the values of primary beams (36, 37). The existing GSI accelerator facility is presented in **Figure 2**.

Due to its dual capability as separator and high-resolution spectrometer, the FRS can be used for production, identification, energy bunching and spatial separation of the secondary beams (in particular of therapy-relevant PET isotopes such as ^10,11^C and ^14,15^O), for tailoring specific phase-space properties of the secondary beams as well as for detailed experimental studies of atomic and nuclear processes, that are of basic and practical interest for heavy-ion therapy and related imaging applications. The FRS provides these possibilities at several experimental areas, for instance at the final focus of its symmetric main branch, where first PET measurements have been conducted (38), or *via* its target-hall branch to the medical Cave M, which was recently commissioned. In Cave M, legal permissions exist to irradiate animals. The connecting branch of FRS to Cave-M allows the transport and delivery of isotopically clean secondary beams like ^11^C or ^15^O with rates ~10^7^ particles per second (pps).

### Basic Atomic and Nuclear Studies

**Figure 3:**
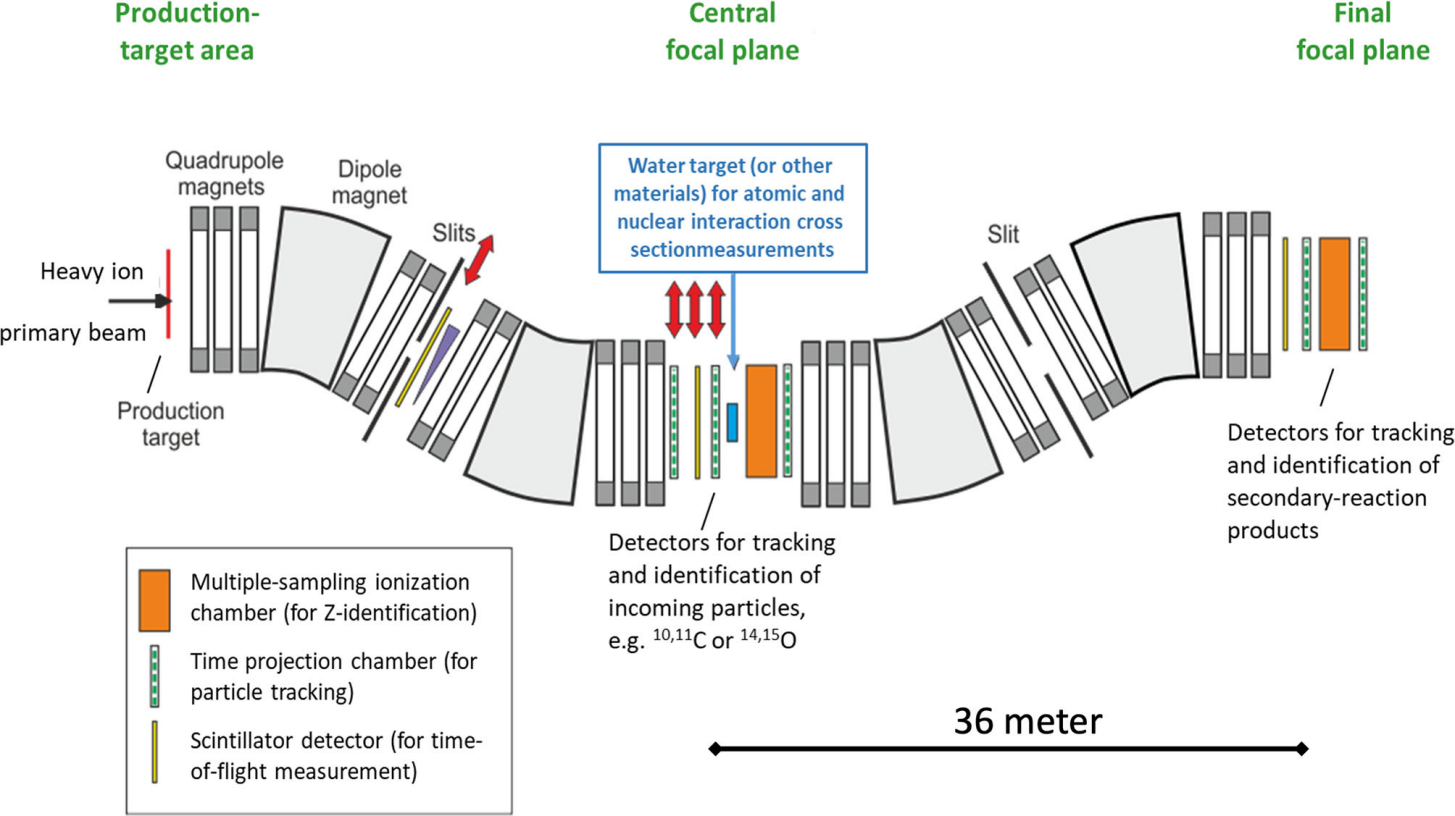
Schematic view of the symmetric FRS branch (projection in the horizontal dispersive plane) and the experimental setup to measure the interaction cross-section and nuclear charge composition of the beam fragmented in water. Red double-sided arrows indicate remotely removable detectors.

The planned basic studies aim at the production yields of isotopes for PET and at a detailed understanding of their atomic and nuclear interactions in matter resp. tissue equivalent materials such as water. For instance, there have been several measurements on the production cross-sections of PET isotopes from stable beams, but the published results widely scatter (39). A systematic investigation of the production cross sections is necessary in order to optimize the yield and the properties of the in-flight separated radioactive ion beams that will be used for mice treatment; for detailed modeling of dose distributions, it will be important to obtain the total interaction and nuclear charge-changing cross sections in the relevant energy regime. The experimental techniques are well established and have been widely used at the FRS (40–43). The isotopes of interest (such as ^10,11^C, ^14,15^O) will be produced, separated and identified with the first half of the separator, impinge on a secondary reaction target located at the middle focal plane, where secondary reactions are induced, and the reaction products will be analyzed and identified using event-by-event information of magnetic-rigidity, time of flight, and energy deposition; the total interaction cross sections will be determined using the number of non-reacted isotopes. A schematic view of the experimental setup is shown in **Figure 3**.

The atomic interaction (energy loss, energy-loss straggling and angular straggling) of the ion-beam with the tissue is the dominant physical process involved in the ion-beam therapy, and the accurate understanding of corresponding properties like range and range straggling are of very high practical importance. At HIMAC (Japan), the ranges of various PET isotopes (^10,11^C, ^14,15^O) have been investigated extensively (27, 44, 45). The range distribution of the selected fragments is primarily determined by the initial energy distribution. The maximum momentum spread of the in-flight separated RIB at the FRS is defined by its longitudinal momentum acceptance, which is approximately ±1%. This momentum spread can be considerably reduced by using a mono-energetic degrader placed at the dispersive focal plane (37), so that the longitudinal range distribution reaches a spread very similar to the range straggling of a primary beam of the same energy.

The latter performance is strongly correlated with the ion-optical resolution (38). Alternatively, by changing the shape of the degrader the momentum distribution can also be increased to achieve a spread-out Bragg-peak (SOBP), if needed. Such studies shall be performed using water phantoms in combination with the University Medical Center Groningen (UMCG) PET camera at the final focus of the FRS (see below).

## Dosimetry

Dosimetry and beam delivery monitoring of RIBs are necessary to correlate the collected PET and prompt gamma signals with dose deposition maps for range verification. Additionally, beam parameters such as divergence, lateral profiles and energy spread are required as input in transport code, treatment planning and radiobiological models. Dosimetry is therefore an essential component of the BARB experimental campaign, necessary for all other imaging and radiobiological endpoints.

**Figure 4:**
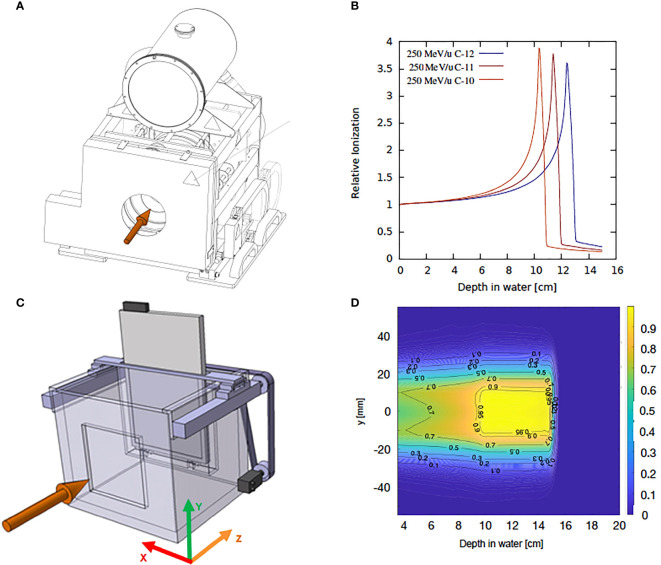
Schematic representation of the experimental setup and typical dose distributions measured with water column and the WERNER setup. **(A)** Technical drawing of the water column. **(B)** FLUKA simulation of laterally integrated 1D depth dose distribution for carbon ion isotopes of 250 MeV/u. **(C)** schematic representation of the WERNER setup. **(D)** 2D dose distribution map for a 4 cm- ^12^C SOBP.

Range monitoring and depth dose distributions of the pristine Bragg peak and SOBP - obtained by using 3D printed modulators (46) ***-*** will be measured using the water column setup shown in **Figure 4** (47). Two parallel plate ionization chambers (ICs) are placed at the two extremes of a water phantom with precisely adjustable thickness. The water phantom thickness is controlled by a stepper motor and can be varied with a relative precision down to 10 µm. The ICs are read out with two Keithley K6517A electrometers. The laterally integrated depth dose distribution is then measured as the ratio between the signals collected by the two ICs as a function of the water depth.

To correlate the dose deposition maps with the acquired PET images and to verify treatment plans delivered with RIB, 3D dosimetry will be performed. For this purpose, the water column setup WERNER (WatER column for 2D ioNization chambEr aRray detectors) (48), which was designed for ion beam therapy applications, will be used. This system consists of a plastic water tank of 40 × 33.5 ×35 cm^3^ where a watertight detector container attached to a stepper motor is placed inside. The detector position can be changed along the beam direction with a precision of about 100 µm. The system is controlled with a LabVIEW‐based control software and synchronized to the beam delivery system. The WERNER detector holder is designed for the PTW 2D IC arrays designed for proton and ion therapy, OCTAVIUS 1500XDR and OCTAVIUS 1000P. The first one consists in a 2D array of 1405 ICs distributed in a chessboard matrix of 27 × 27 cm^2^. The center-to-center distance between two ICs is about 7.1 mm and the dose resolution is 0.1 mGy. The OCTAVIUS 1000P is a detector prototype consisting of 977 ICs of 2.3 × 2.3 × 0.5 mm^3^ volume with a spatial resolution of 2.5 mm in the 5.5 × 5.5 cm^2^ central area and 5 mm in the 11 × 11 cm^2^ outer area. A schematic of the WERNER setup and a typical dose map are depicted in **Figure 4**. Additionally, beam profiles and beam divergence will be measured with high spatial resolution Gafchromic^®^ EBT films (International Specialty Products, Wayne, NJ) free in air or a stack of films interlaced with plastic absorbers. An accurate calibration of the beam delivery system in terms of fluence - and thus dose- with the precision standards required for therapy applications will be achieved following the standard GSI protocol for beam monitoring chamber calibration (49).

## UMCG PET

**Figure 5:**
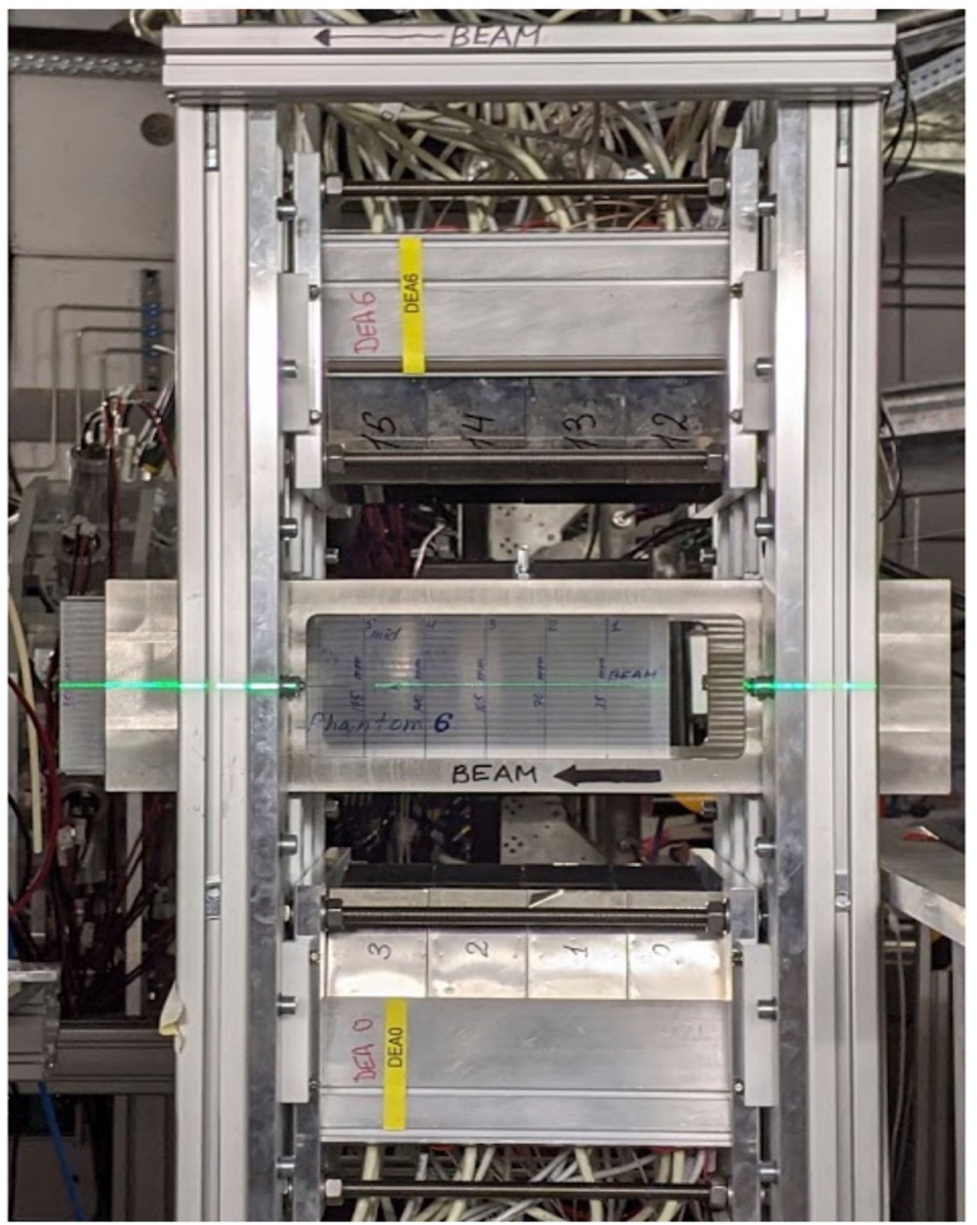
The dual-panel UMCG positron imaging system installed at the Fragment Separator FRS at GSI. A beam of radioactive ions is coming from the right hand side and stopped in the PMMA phantom seen in the middle of the picture. The two detector panels are installed above and below the phantom each at a distance of 30 cm.

The dual-panel positron imaging system of the University Medical Center Groningen (UMCG) is 1/6 of a Siemens Biograph mCT clinical positron emission tomography (PET) scanner (50) with custom-modified detectors. The two detector panels are installed opposite to each other, typically at a distance of 25-30 cm. The phantom in which the beam is stopped is placed in-between the panels (**Figure 5**).

Being designed for a ring-shaped scanner with a detector ring diameter of 84.2 cm, the detector panels are curved with a radius of curvature of 42.1 cm in one direction and are flat in the other, perpendicular, direction. Each panel covers an area of 22.0×22.5 cm^2^ and is composed of a 4×4 array of block detectors. A block detector comprises a 13×13 array of 4×4×20 mm^3^ LSO scintillation crystals read out by 4 photomultiplier tubes (PMTs). Anger logic performed on the 4 PMT signals from one event enables to identify the scintillation crystal in which the gamma ray interaction took place. The detectors have been custom-modified such that they can be switched off and on with switching times of less than a millisecond. This switching has been essential in earlier work on nitrogen-12 positron imaging in proton and helium therapy, where short and intense beam pulses were used (51, 52). Whether this option will be useful when imaging radioactive beams will depend on the time structure and intensity of the beams.

Each time a valid coincidence between the two panels is detected, the listmode data acquisition registers which scintillation crystals are involved, the coincidence time (the time difference between the gamma ray detection by the 2 detectors) as well as a clock time stamp with an accuracy of 1 ms. A coincidence is valid if the energy detected is within a user-defined energy window around 511 keV, typically 435-650 keV, and the coincidence time is within the user-defined coincidence time window, typically 4 ns. Knowing which scintillation crystals were involved in a coincidence enables to establish the Line-of-Response (LoR) as the line connecting the two crystals. The LoR’s are subsequently used to generate an image. The 550 ps (FWHM) time-of-flight (ToF) resolution of the system restricts the position of positron annihilation along a LoR of about 8 cm (FWHM). The ToF information will be useful to improve the contrast-to-noise ratio of the images in case the size of the irradiated area is comparable to or larger than this 8 cm.

## LMU Detector

**Figure 6:**
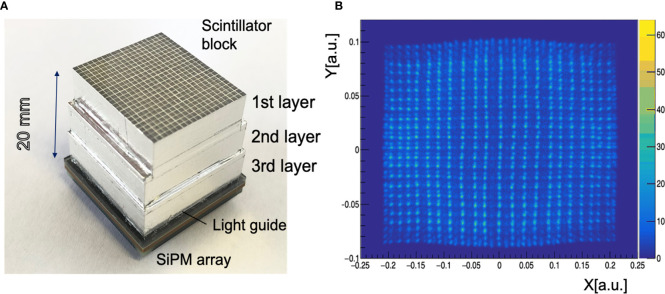
LMU hybrid γ-PET detector **(A)** A 3-layer PET detector developed at LMU Munich in collaboration with NIRS-QST. The PET detector consists of a 3-layer scintillator block, a light guide and an 8 × 8 SiPM array. **(B)** A flood map of the 3-layer PET detector exposed to a ^22^Na radioactive point source.

The hybrid detector able to visualize PET, PG and even triple coincidence emissions (e.g., from ^10^C) for the BARB project is going to be developed at LMU by combining detector technologies currently under investigation for PET and PG (7) imaging, as well as a combination thereof in the context of proton therapy (53). In particular, the absorber component of the envisioned hybrid BARB detector will rely on a high resolution PET detector which was recently developed at LMU, in collaboration with NIRS-QST, for an in-beam small animal PET scanner prototype within the ERC-funded project “Small Animal Proton Irradiator for Research in Molecular Image-guided Radiation-Oncology” (SIRMIO) (10). This LMU-PET detector aims to achieve sub-millimeter spatial resolution along with the capability of identifying the Depth-Of-Interaction (DOI) (54). The latter DOI information reduces the effect of parallax errors, which cause degradation of a PET image at the peripheral areas of a field of view. **Figure 6A** shows the LMU-PET detector, which is composed of a 3-layer Lu_1.9_Y_0.1_SiO_5_(LYSO, density: 7.25 g/cm^3^) scintillator block (EPIC, China) and a SiPM array. The scintillator pixel size is 0.9 mm × 0.9 mm × 6.67 mm. The 1^st^, 2^nd^ and 3^rd^ layers consist of arrays of 23 × 20, 23 × 23 and 24 × 24 pixels, respectively, to form a staggered DOI detector. An 8 × 8 multi-pixel photon counter array (MPPC, micro-cell size: 50 μm × 50 μm, each sensitive area: 3 mm × 3 mm, total area: 25.8 mm × 25.8 mm, Hamamatsu photonics K.K, S14161-3050HS-08, Japan) is used as photo detector. A light guide with a thickness of 1 mm is inserted between the 3-layer scintillator block and the MPPC array. We use a charge division circuit to reduce the 64 signals from the MPPC array to 4 single-ended readouts (55). The 4 single-ended readouts are processed by an amplifier circuit board and converted to a differential signal. The differential signals are fed to a digitizer (R5560, CAEN, Italy). An Anger calculation is used to project an interaction position between the scintillator pixel and a γ-ray on a 2-D position histogram. The Anger calculation result of the scintillator pixel forms a pixel response as a cluster in the 2-D position histogram, which is called a flood map. Because each pixel response is drawn without overlapping each other, the interaction position can be identified. **Figure 6B** shows the flood map obtained by irradiating 511 keV annihilation γ-rays from a ^22^Na point source. Pixel responses of each layer are clearly separated, indicating the pixel resolution of 0.9 mm.

### BARB4D

The treatment of targets in intrafractionally moving organs poses a significant challenge (56), not only in conformal delivery strategies (57), but especially also in visualizing complex motion within the patient (58). The workhorse for current 4D-treatment planning is time-resolved computed tomography (4DCT) in combination with Deformable Image Registration (DIR). Both have caveats: the 4DCT depicts a single synthesized breathing cycle, and DIR has known inaccuracies and is hard to verify, especially in organs that offer low contrast in the CT, such as the liver or heart.

PET offers an exciting opportunity to study 4D-dose deposition in complex geometries or patients. Time-resolved PET imaging has the advantage of showing the deposited activity, which moves with the respective organ, following also complex deformations or rotations not visible with other methods. Previous research (13, 59) was hampered by low activity and the dissociation of dose and activity, both of which can be resolved with RIBs.

**Figure 7:**
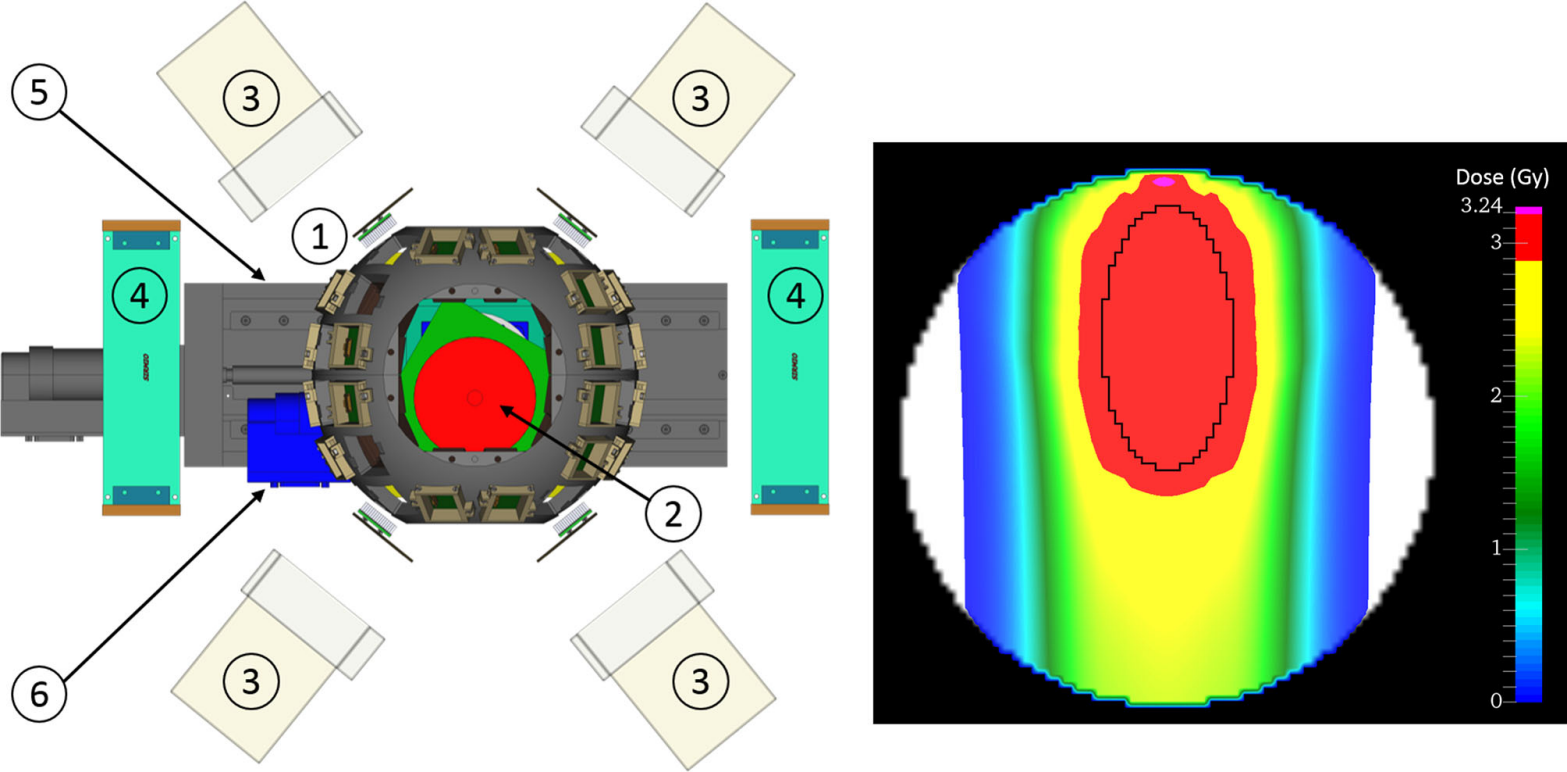
The BARB4D setup: (1) SIRMIO cage with 32 PET detectors, (2) cylindrical phantom, (3) Compton camera, (4) Amplifier circuit boards for PET detectors, (5) translation stage, (6) rotating stage. The beam is entering the phantom from below. Right: Planned dose distribution for a static delivery with ellipsoidal target region.

We propose a proof-of-concept study in small, rotating phantoms that both fit into the small Volume-Of-Interest (VOI) of the planned BARB detector and still offer a significant motion amplitude (**Figure 7**). High-resolution PET will resolve both uncompensated interplay distortion of dose and the efficacy of motion mitigation strategies in this complex scenario. Ideally, experiments would continue in a larger animal model, which permits to study realistic complex motion patterns that are difficult to simulate in phantoms. In this way, time-resolved RIB PET provides an endpoint to test motion detection and DIR as well as 4D-dose reconstruction and motion mitigation strategies. This work could be continued in a later clinical facility with human patients, but will already provide valuable input for 4D-delivery research in the project lifetime of BARB.

## Radiobiology

The final goal of BARB is a tumor treatment in an animal model with RIBs. This will be the final proof of the potential of RIBs in particle therapy, and will assess the real advantages compared to stable ions. The RIB will be then directed to biological targets, first *in vitro* mammalian cell cultures and finally a mouse tumor.

### *In Vitro* Experiments

**Figure 8:**
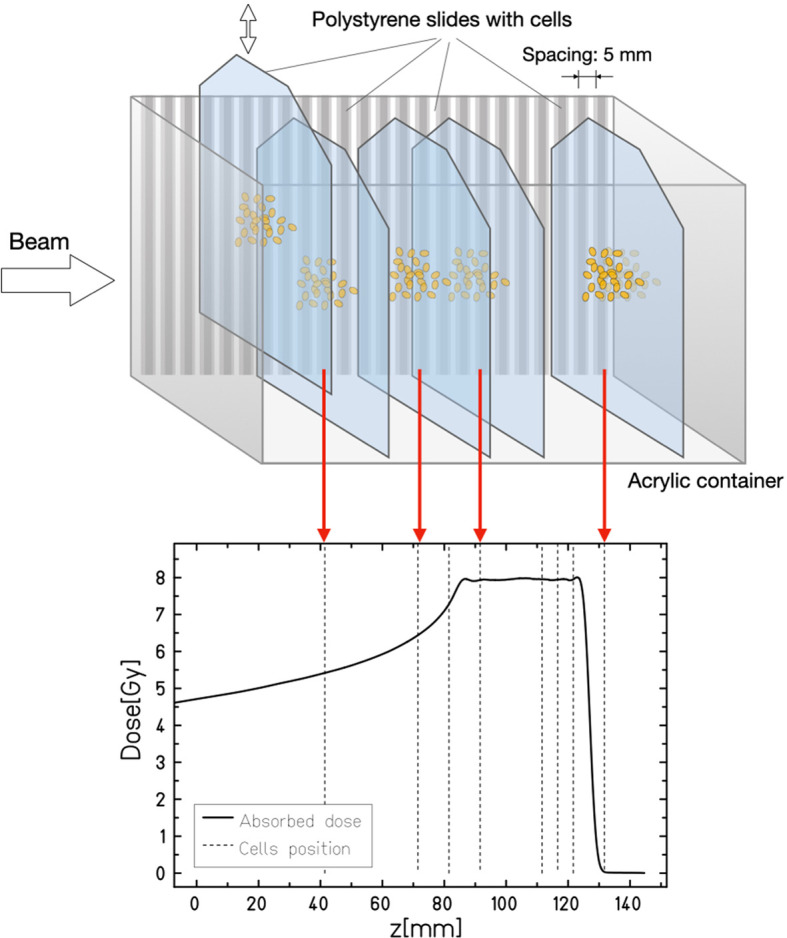
Cellular phantom used for radiobiological measurements along a SOBP. The cells grow in monolayer on plastic plates that can be plunged at different position in the tank filled with water-equivalent growth medium. Plates are then removed after irradiation and the cell survival is measured in every position. In the BARB experiment, stable and radioactive carbon and oxygen isotopes will be used to irradiate CHO cells, under the null-hypothesis that no difference will be observed.

Measurement of the Relative Biological Effectiveness (RBE) is essential in heavy ion therapy, because the RBE varies along the Bragg peak and can be high in the distal part (60). The Local Effect Model (LEM), coupled to the deterministic transport code TRiP98, is used in the European clinical centers for treatment planning (61). In fact, LEM reproduces very well the survival of mammalian cells to both carbon (62) and oxygen beams (63). LEM assumes that the RBE depends on the charge and velocity of the ion, so no significant differences are expected between radioactive isotopes and stable ^12^C and ^16^O ions. Similar RBE values for stable and radioactive light ions at different depths in the spread-out-Bragg-peak (SOBP) are also predicted by the microdosimetric kinetic model (64, 65). However, models are affected by large uncertainties (66, 67) and differences may be caused by the different nuclear interactions and the production of secondary particles. We will therefore repeat the dosimetry experiments using a cellular phantom (62), where cell killing can be accurately measured at different depths along the SOBP (**Figure 8**). Results of the survival curves in Chinese Hamster Ovary (CHO) cells at different positions will be compared to the TriP98/LEM predictions.

### Animal Experiments

The final test of our method will be the first ever treatment of a tumor by RIB. We will use a mouse model, which can be visualized with the small Filed-Of-View (FOV) of our hybrid detector (see *LMU Detector*). We will focus on an orthotopic mouse model and, for comparison, a xenograft. Syngeneic allografts will be prepared for abdominal tumors in nude mice, while xenograft will be implanted in immune-competent strains.

**Figure 9:**
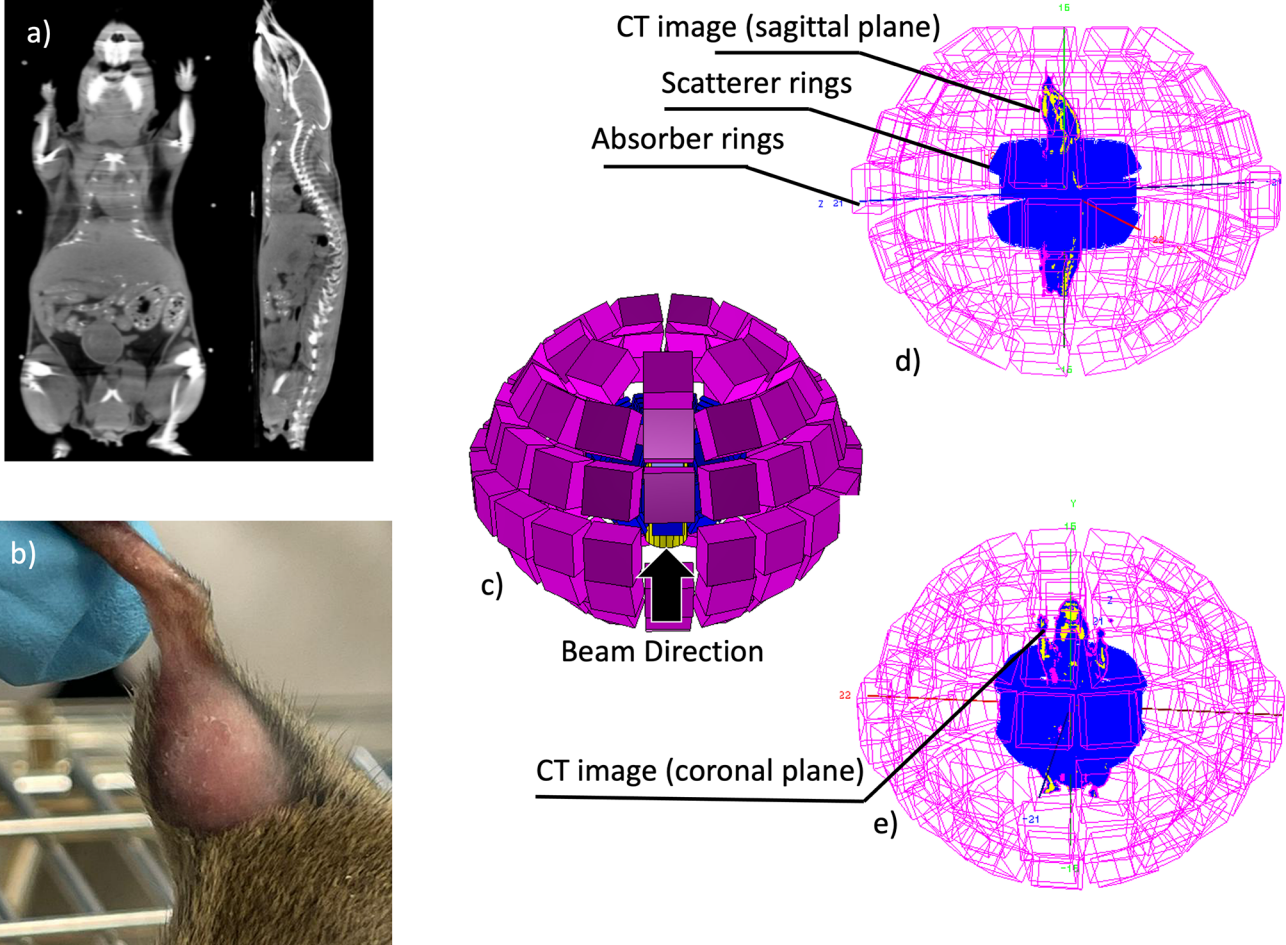
**(A)** Mouse CT image, coronal and sagittal planes, **(B)** example of an osteosarcoma in the C3H mouse hind limb; **(C)** the proposed hybrid Compton-PET scanner for the radioactive ion beam range verification, **(D, E)** the scanner configuration with the CT image of the mouse (sagittal and coronal planes are plotted, respectively) positioned along the scanner bore.

We have previously worked on an autochthonous model of murine soft tissue sarcoma in the mouse leg (68). In that experiment, the tumors were irradiated with a 3-cm SOBP from a 110 MeV/u ^12^C beam in the whole leg (69). With similar large fields, we have irradiated LM8 osteosarcomas in the hind limb of C3H mice (**Figure 9**) with C-ions (70). In fact, control of the beam in the small mouse tumors would be very difficult without online imaging. Here we aim to reduce the margins to show that we can precisely irradiate small murine tumors whilst sparing the surrounding normal tissue. The choice of the RIB isotope will come from the output of the experiments in *Dosimetry*. A single dose will be used, and the RIB physical dose in Gy will be corrected for the RBE, based on the results in *In Vitro Experiments*, in order to compare equally effective doses. For the orthotopic model, we will plan the mouse treatment based on µCT, available in our experimental room Cave M at GSI, and will apply a very small margin of approximately 0.5 mm for a tumor, whose diameter will be approximately 5-6 mm. The µCT data can be imported into the MEGAlib geometry file (71) of the SIRMIO hybrid detector (10), thus allowing full Monte Carlo simulation of the experiment (**Figure 9**). We will irradiate anesthetized and immobilized mice with a single beam port, a situation where range uncertainty is critical. The hybrid detector prototype for small animals will be used for online monitoring of both stable and radioactive beams. Tumor growth will be measured every three days. Animals will be finally sacrificed 21 days post-exposure and following a final CT scan. Histological analysis will be used to determine the irradiated tissue. Our working hypothesis is that the improved accuracy with RIB translates into improved local control compared to stable ion treatments with small margins that may miss the target. These experiments will provide the best accuracy achievable *in vivo* with RIB and the impact of the improved precision on the control of small tumors.

### RIB as *In Vivo* Tracers

Additional experiments will be performed using RIBs as radioactive tracers implanted in the tumors. The purpose will be to clarify the role of vascular damage in single-fraction high-dose radiotherapy (72). One hypothesis of the clinical success of single-dose radiotherapy (73), even compared to hypofractionation (74), is the vascular dysfunction (75) *via* ischemia/reperfusion injury (76). Other authors contend that the increased effectiveness of single-fraction is well explained by the classical radiobiology in terms of reduced repair, i.e. increased biologically effective dose, and no special role of the vascular system is necessary to explain the clinical results (77, 78). A recent study using dynamic contrast-enhanced magnetic resonance imaging in a rat tumor model showed that high doses of X-rays or C-ions enhance vascular damage and increase permeability of the tumor (79, 80). We have the opportunity of using the RIB as very precise monitor of the vascular permeability, because essentially we deposit a high concentration of radiotracers in the tumor in a very short time. By measuring in PET the washout of the signal with the arrangement shown in **Figure 9**, corrected for the physical half-life after low and high doses, we will assess the different vascular permeability at different doses and will assess whether at doses higher than a threshold this mechanism can lead to tumor control. This will clarify whether vascular damage plays a role in high-dose single-fraction.

## Conclusions and Outlook

**Figure 10:**
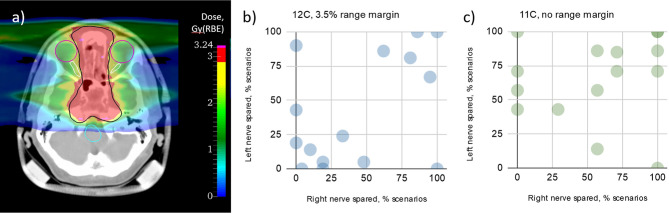
Impact of range margin reduction in robust optimization on the optic nerves sparing for a set of ACC patients. **(A)** Example of CT and two-field dose distribution for one of the analyzed patient plans. CTV, optic nerves, eyes and brainstem are contoured with black, white, purple and turquoise colors, respectively. Color bar represents the dose distribution (prescribed dose 3 Gy(RBE)). Primary goal of the robust optimization was achieving at least 95% of the prescribed dose in 95% of uncertainty scenarios (21 scenarios in total). Graphs represent sparing of left and right optic nerves in the analyzed plans with ^12^C beams **(B)** (3.5% range and 3 mm setup margin) and ^11^C plans **(C)** (3 mm setup margin only), respectively. Each point corresponds to a single patient case (15 patients total).

For many years, RIBs have been proposed as the ideal bullet for image-guided particle therapy (29). The practical advantage of RIB therapy compared to conventional stable-ion treatments remains howbeit unproven. The theoretical advantage can be estimated with a treatment planning calculation of the dosimetric advantage gained by reducing the margins (81). **Figure 1** suggests that beam visualization with RIB can essentially eliminate the range uncertainty (3.5% of the range), leaving only the setup margin. We applied this concept to the patients treated at GSI during the pilot project for adenoid cystic carcinoma (ACC) with a boost of carbon ions after intensity-modulated radiotherapy in Heidelberg (82, 83). We have re-evaluated the C-ions treatment plans using robust planning and looking at the potential reductions in normal tissue toxicity when the clinical target volume (CTV) margin is reduced to the re-positioning uncertainty (3 mm) only. Tolerance of the optic nerve was set according to the recommendations of the European Particle Therapy Network as D_0.03 cc_<55 Gy and α/β= 2 Gy (84). As shown in **Figure 10**, we found that margin reduction using RIB leads to a significant sparing of the optical nerves in more than 50% of the patients.

The BARB project will therefore clarify the real advantage of RIB therapy, reaching the stage of the treatment of an animal patient with ^11^C and ^15^O and simultaneous beam visualization. BARB will exploit the intensity upgrade in FAIR-phase-0 and a novel γ-PET detector for beam visualization. It can be contended that even positive and exciting results will hardly have clinical impact, because in-flight production of RIBs would be impractical in current medical synchrotrons. However, already during the pilot heavy ion project at the Lawrence Berkeley Laboratory (CA, USA), it was proposed to produce the RIBs at low energy and then inject them in the high-energy medical accelerator (85). The idea is to build a small cyclotron that can produce low-energy RIBs with an ISOL system (86), and these ions are then injected in conventional synchrotrons. A source using low-energy electron beams for the production of ^11^C has been designed and produced at HIMAC (87). Within the MEDICIS-Promed project (88), CERN has proposed a charge breeding scheme based on an Electron Beam Ion Source for beam preparation of a radioactive ^11^C beam (89). The charge breeder is coupled to a medical synchrotron currently used for ^12^C-ion therapy to treat patients with ^11^C using the same beam delivery devices of conventional heavy-ion therapy (90). The future of this ambitious project will depend on the results of the BARB project in the coming five-year period.

## Data Availability Statement

The raw data supporting the conclusions of this article will be made available by the authors, without undue reservation.

## Author Contributions

The authors of this manuscript are all working in the BARB experiment. The paper was read and discussed among different authors. Some lead author took the responsibility to write the different sections: MD drafted sections 1, 6, and 7; CSche drafted section 2; DB and UW drafted section 3; PD drafted section 4; KP, MN, and CGr drafted section 5. All authors contributed to the article and approved the submitted version.

## Funding

This work is supported by European Research Council (ERC) Advanced Grant 883425 BARB to MD and in part by the ERC Consolidator Grant 725539 SIRMIO to KP. The measurements described here are performed within the experiments S533_Purushothaman and SBio08_Parodi at SIS18/FRS/S4/Cave-M at the GSI Helmholtzzentrum für Schwerionenforschung, Darmstadt (Germany) in the frame of FAIR Phase-0.

## Conflict of Interest

Author AP was employed by company MedAuston GmbH.

The remaining authors declare that the research was conducted in the absence of any commercial or financial relationships that could be construed as a potential conflict of interest.

## Publisher’s Note

All claims expressed in this article are solely those of the authors and do not necessarily represent those of their affiliated organizations, or those of the publisher, the editors and the reviewers. Any product that may be evaluated in this article, or claim that may be made by its manufacturer, is not guaranteed or endorsed by the publisher.
